# Analytical validation of GenoPharm a clinical genotyping open array panel of 46 pharmacogenes inclusive of variants unique to people of African ancestry

**DOI:** 10.1371/journal.pone.0292131

**Published:** 2023-10-03

**Authors:** Comfort Ropafadzo Kanji, Bianza Tinotenda Mbavha, Collen Masimirembwa, Roslyn Stella Thelingwani

**Affiliations:** 1 Department of Genomic Medicine, African Institute of Biomedical Science and Technology (AiBST), Beatrice, Zimbabwe; 2 Department of Clinical Pharmacology, University of Zimbabwe (UZ), Harare, Zimbabwe; 3 CradleOmics, Harare, Zimbabwe; Jordan University of Science and Technology, JORDAN

## Abstract

Pharmacogenomic testing may be used to improve treatment outcomes and reduce the frequency of adverse drug reactions (ADRs). Population specific, targeted pharmacogenetics (PGx) panel-based testing methods enable sensitive, accurate and economical implementation of precision medicine. We evaluated the analytical performance of the GenoPharm^®^ custom open array platform which evaluates 120 SNPs across 46 pharmacogenes. Using commercially available reference samples (Coriell Biorepository) and in-house extracted DNA, we assessed accuracy, precision, and linearity of GenoPharm^®^. We then used GenoPharm^®^ on 218 samples from two Southern African black populations and determined allele and genotype frequencies for selected actionable variants. Across all assays, the GenoPharm^®^ panel demonstrated 99.5% concordance with the Coriell reference samples, with 98.9% reproducibility. We observed high frequencies of key genetic variants in people of African ancestry: CYP2B6*6 (0.35), CYP2C9*8, *11 (0.13, 0.03), CYP2D6*17 (0.21) and *29 (0.11). GenoPharm^®^ open array is therefore an accurate, reproducible and sensitive test that can be used for clinical pharmacogenetic testing and is inclusive of variants specific to the people of African ancestry.

## Introduction

In the emerging practice of genomics-guided precision medicine, pharmacogenetics has been identified as low-hanging fruit to demonstrate clinical utility. Pharmacogenomics takes observed variability into account and describes how to use genetic information for the safe and efficacious use of medicine. Clinical pharmacogenomic (PGx) testing for drug-gene pairs with actionable practice guidelines has been widely recognized as a tool for guiding clinicians in selecting drugs and drug doses predicted to result in optimal treatment outcomes [1, 2]. Single nucleotide polymorphisms (SNPs) in drug metabolising enzymes confer different activities and functionality of enzymes ranging from normal metabolizers (NM), intermediate metabolizers (IM), poor metabolizers (PM), and ultra-rapid metabolizers (UM) [3]. When these genetic variations affecting drug pharmacokinetics are combined with variants affecting drug effect processes, or pharmacodynamics, clinical outcomes can be influenced. For example, warfarin has genes that affect both the pharmacokinetic (PK) pathway and pharmacodynamic (PD) processes [4].

Reactive and pre-emptive testing have been the two main approaches in PGx testing. The reactive model is guided by evaluation at the time of prescription, emerging or past adverse drug events, and/or lack of efficacy. Pre-emptive testing addresses potential therapies without considering medical history or past medication. This has made multiplex genotyping panels a standard for pre-emptive testing, especially in primary and secondary care settings. Other platforms, such as whole genome sequencing, have also been utilised, especially in tertiary care settings. However, the use of pharmacogenetics in the clinical setting has been slow. Increasing efforts have seen PGx information ranging from cautionary statements to clinical guidelines for over 200 medicines on the market [5]. This has spurred the development of multiplex genotyping tools to support clinical PGx deployment, starting with the FDA approved AmpliChip from Roche [6]. It has also led to the development and validation of specific PGx testing panels targeting either a disease or population [7–10].

Genetic variants in drug metabolising enzymes, transporters, receptors, and other proteins involved in drug response vary widely across different populations [11]. This is where targeted genotyping falls short, as it may miss population-specific variants which in turn affects the specificity and sensitivity of genotyping assays. CYP2D6 is one of the most studied cytochrome P450 (CYP) enzymes, which shows variability with marked population differences. For example, CYP2D6*4 frequency is high in European (22%) and Jewish (18%) populations but has a lower frequency in Asians (0.6%) and Black Africans (2%) [12]. The CYP2D6*10 is common in Asians (41%) [13] while the *17 and *26 are common in African populations at a frequency of 34% and 20% respectively [14, 15]. This has implications on drug response and risk to adverse events in the different population groups. Pharmacogenetic based guidelines to guide the use of medications have been published [16]. Surveillance has shown that more than 90% of individuals carry genes that put them at risk if they take medications described in the guidelines. The risk is, however, preventable if proper guidance is sought through pharmacogenetic testing.

There are a number of commercially available pharmacogenetic testing panels but the published literature on analytical validation is limited. Extensive evaluation of the analytical performance of genotyping assays is important to strengthen the quality of pharmacogenetic data. Before a test is used to support patient care, it needs to be validated. Clinical Laboratory Improvement Amendment (CLIA) states that the test should be validated for performance characteristics which include accuracy, precision, reportable range, reference interval, analytical sensitivity, and analytical specificity [17]. We have developed a custom open array panel, the GenoPharm^®^ test panel, which is an open array that captures genes for drug-gene pairs, inclusive of those for which there is evidence that supports therapeutic management recommendations (Table 1). In this paper, we describe the analytical validation of the custom open array.

**Table 1:** Genes and variants included in the GenoPharm^®^ pharmacogenomic panel.

**Phase I ADME genes**	
ADLH2: Glu504Lys (rs671)	CYP2B6: (*4, *5, *6, *18)
CYP3A4: *1B, *22	CYP1A2: (*1C, *1D, *1E, *1F, *1K, *1V)
CYP3A5: *3, *6, *7	CYP2C19: (*2, *3, *4, *7, *10, *17)
CYP4F2: *3	CYP2C9: *2, *3, *4, *5, *6, *8, 11, C.1425A>T (rs1057911)
DPYD: *2A, *13, c.2846A>T	CYP2D6: (*2, *2A, *4, *6, *7, *8, *9, *10, *11, *12, *14, *15, *17, *18, *19, *29, *31, *35, *41, *42, *44, *59, 4180G>C (rs1135840)
**Phase II ADME genes**	
COMT: Val158Met (rs4680)	NAT2: *5, *6, *7, *11, *12, *13, *14
UGT1A6: *4 (rs17863783)	UGT1A1: *6 (rs4148323), -2936A>G (rs4124874)
TPMT: *2, *3C, 3B, *4	SULT4A1: rs138097, rs138060
**Transporters**	
ABCG2: c.421C>A (rs2231142)	ABCB1: c.1236T>C (rs1128503), c.2677T>G/A (rs2032582),
SLC28A3: L461L (rs7853758)	c.3435C>T (rs1045642)
SLC2A2: T110I (rs5400)	SLCO1B1: *1B, *5, *15, *17, *21
**Drug Target**	
VKORC1: *2, *3F	
**Other pharmacokinetic and pharmacodynamic genes**	
ACE: G2350A (rs4343)	CACNA1C-AS2: rs1051375
ADRA2A: C1291G (rs1800544	CDKN2B-AS1: rs1333049, rs10757278 ACD risk
AGT: A-6G (rs5051)	G6PD: 376A>G (rs1050829)
BDNF: G196A (rs6265)	GRIK4: 83-10039T>C (rs1954787)
CELF4: rs1786814	HLA-B: *1502, *1502, *5801, *5801
DRD2: -241A>G (rs1799978)	LPA: I4399M (rs3798220), rs10455872
FABP2: Ala54Thr (rs1799883)	HTR2C: -759C/T (rs3813929)
F2: G20210 (rs1799963)	MTHFR: c.1298A>C (rs1801131), c.677C>T (rs1801133)
F5: R506Q (rs6025)	NUDT15: c.415C>T (rs116855232)
GRIN2B: rs2058878	OPRM1: c.118A>G (rs1799971)
HTR2A: rs7997012	RARG: S427L (rs2229774)
IFNL3 (IL28B): (rs12979860)	VEGFA: -634C>G (rs2010963)

## Materials and methods

### Panel design and selection of variants

Selection of the genes in the panel was based on literature review. We selected 43 genes which have been described to have pharmacokinetic or pharmacodynamic associations. Twenty six of the genes have gene-drug associations for PharmGKB top four highest levels of clinical significance (level 1A, 1B, 2A and 2B). For CYP2D6, CYP2C9 and DPYD we included all the recommended variants within the Association for Molecular Pathology (AMP) tier 1 group. The genes include those with Clinical Pharmacogenetics Implementation Consortium (CPIC), the Royal Dutch Association for the Advancement of Pharmacy—Pharmacogenetics Working Group (DPWG), the Canadian Pharmacogenomics Network for Drug Safety (CPNDS) dosing guidelines (https://www.pharmgkb.org/guidelineAnnotations).

We chose to include variants based on their Minor Allele Frequencies (MAF), to include variants specific to the African population. In total the GenoPharm^®^ panel consists of 120 genetic variants of 46 genes that have been uniquely selected/composed to predict and guide patient treatment needs in general and specifically for infectious diseases, cancer, cardiovascular disease, and neuropsychiatric disorders. The panel was designed to enable high throughput screening to simultaneously process many samples across a number of targets. The kit is currently registered and licenced for use in Zimbabwe by the Medicines Control Authority of Zimbabwe (MCAZ) and in South Africa by the South African Health Products Regulatory Authority (SAHPRA).

The GenoPharm^®^ open array is composed of two testing components, one for SNP genotyping and the other for *CYP2D6* copy number variation (CNV). The genotyping component consists of 120 TaqMan^™^ assays for 118 loci (Table 1), which include single base and short insertion/deletion polymorphisms. Selected assays include 11 phase I drug metabolising enzymes (7 of them belonging to the Cytochrome P450 family), 6 phase II enzymes and 5 drug transporters single nucleotide polymorphisms (SNPs) (Table 1). These assays identify alleles, in human samples obtained from genomic DNA extracted from either whole blood or buccal swabs, which are used to determine drug metabolism, drug response, and specific disease risk factors. The TaqMan^™^ Copy Number Assay examines the copy number variations in exon 9 of the CYP2D6 gene (Table 2), which helps to further define drug response.

**Table 2:** Copy number variation assays and controls for CYP2D6 gene targeting exon9.

Coriell ID	Predicted GT	Observed Copy number
NA14476	CYP2D6 no CNV	2
NA17104	CYP2D6 duplication	3
NA17105	CYP2D6 duplication	3
NA17107	CYP2D6 deletion	1
NA17109	2 copies CYP2D6 + CYP2D6*36	2
NA17112	CYP2D6 no CNV	2
NA17114	CYP2D6 deletion	1
NA17116	CYP2D6 no CNV	2
NA17117	CYP2D6 duplication	3
NA17118	CYP2D6 no CNV	2
NA17128	CYP2D6 no CNV	2
NA17155	CYP2D6 duplication	3
NA17209	2 copies CYP2D6 + CYP2D6*36	2
NA17232	CYP2D6 duplication	3
NA17245	CYP2D6 no CNV	2

### Sample collection and DNA preparation

In this study, we used 43 commercial reference samples and 8 plasmid control samples obtained from the NIGMS Human Genetic Cell Repository at the Coriell Institute for Medical Research (S1 Table in S1 File). In addition, we had 5 de-identified archived samples as controls for variants which did not have commercial reference sample. We tested 218 individual archived samples from participants in a black Zimbabwean healthy population (n = 118) and black female South African ER+ breast cancer patient cohort (n = 100). All the samples had written consent for further studies and long term storage. The study protocols for the studies where samples were obtained were approved by the Medical Research Council of Zimbabwe (MRCZ/A/2386), Medicines Control Authority of Zimbabwe (CT170/2018) and the Witwatersrand Human Research Ethics Committee with Clearance Certificate Number M180865. The samples were retrieved from archived study samples collected in the period between 2019 and 2021. Genotyping assays were conducted in 2022. The authors did not have access to information that could identify individual participants during or after data collection. Ethical approval for the current study was waived by the Medical Research Council of Zimbabwe (MRCZ/05/2023).

DNA was extracted from peripheral blood collected in EDTA blood tubes using the Applied Biosystems^™^ MagMAX^™^ DNA Multi-Sample Ultra 2.0 Kit. The automated process was performed on the Thermo Scientific^™^ KingFisher^™^ Flex^™^ Magnetic Particle Processor system which utilizes functionalized magnetic beads. Approximately 50 μg of DNA was prepared from 200 μl of whole blood, according to manufacturers instructions. Extracted DNA samples were quantified by Qubit 4 Fluorometer and normalised to 5 ng/μl for use in copy number variation experiments. DNA samples were stored at -20°C for short term storage or at -80°C for long term storage until genotyping analysis.

### Genotyping

The GenoPharm^®^ open array panel uses TaqMan Assay chemistry for the polymerase chain reaction (PCR) using the Applied Biosystems^™^ QuantStudio^™^ 12K Flex Real-Time PCR System (Applied Biosystems, Singapore). DNA samples were diluted to 50 ng/μL using nuclease-free water (Ambion^®^ Cat No. AM9930) and added to a 384 well plate. A reaction mixture of 5 μl genomic DNA and 5 μl of TaqMan^™^ Genotyping master mix (Cat. No. 4462164) was prepared in a 96-well plate. The plate was covered with adhesive PCR Foil (Thermo Fisher) and centrifuged for 1 min at 500g. The mixture was transferred to the GenoPharm^®^ open array panel using the automated Applied Biosystems^™^ QuantStudio^™^ 12K Flex OpenArray^™^ AccuFill^™^ System according to manufacturer’s instructions. A no template control (NTC; reaction mixture with all reagents but no template DNA) was included in each run. The reaction mix of 33nl of DNA per data point was run on the Applied Biosystems^™^ QuantStudio^™^ 12K Flex Real-Time PCR System. Genotypes for the samples were determined by the TaqMan^™^ Genotyper Software as per manufacturer’s instructions. All calls were made at cycle 40 using the default quality value of QV ≥ 0.95 to assign a genotype call. Samples were run in triplicate.

### Sequencing of reference samples

DNA of the reference samples was sequenced to obtain expected values for the extracted human samples. Sanger sequencing was performed by NeoGenomics (Fort Myers, USA), a CLIA certified laboratory, using the Thermofisher 3730xl platform.

### CYP2D6 copy number variation analysis

CYP2D6 Copy number was determined using the Applied biosystems TaqMan copy number assays for Exon 9 the primary copy number assay (Assay ID: Hs00010001_cn). Exon 9 copy number assay was used to quantify CYP2D6 duplications and identify CYP2D6 gene deletion (*5) in the samples. A duplex real time PCR reaction was done with each run. The duplex run had the human TaqMan Copy number reference assay RnaseP (Cat. No. 4403326) as the reference gene. The copy number assays for each sample including the no template control (NTC) were run in quadruplicate in a 96 well plate normal reaction. Each reaction had total volume of 20 μl with 4 μl of 5 ng/ml normalized gDNA, 10 μl of TaqPath Proamp^™^ master mix (Cat. No. A30866) and the reference assay. The qPCR was run on the QuantStudio 12K Flex real time PCR system. The thermal cycling conditions were in brief, initial denature/ enzyme action at 95ºC for 10 minutes, followed by 40 cycles of denature at 95ºC for 15 seconds and annealing and extend at 60ºC for 60 seconds. The relative quantification of CYP2D6 copy numbers were determined using CopyCaller^®^ Software v2.1 (Life Technologies) software. The copy number was determined based on relative quantification analysis of a comparative cycle threshold (CT) between the unknown sample and RnaseP. In brief, a baseline subtracted cycle threshold (CT) or delta CT (ΔCT) is determined for the two assays. The ΔCT of the RnaseP is subtracted from the ΔCT of the unknown sample, resulting in a (ΔΔCT) and from this calculation, the sample copy number is predicted. By visual inspection of the calculated copy number data, successful calls were determined based on a confidence value of >0.95 and a z score of <1.75. Four replicates were run for each sample.

### Validation

The qualitative accuracy, qualitative reproducibility, minimum concentration/dynamic range (LOD) and qualitative specificity were used as performance characteristics to validate the GenoPharm^®^ genotyping and CNV testing panel. All data was examined and where appropriate manual calls were made during this validation. Quality control samples were used to determine whether an unsuccessful (undetermined) call or an incorrect call can be corrected with an actual value based on clustering pattern of that sample.

#### Qualitative accuracy

The accuracy of the GenoPharm^®^ test was evaluated using Coriell reference samples with known genotypes. The reference samples have variants of clinical importance and have been confirmed by multiple volunteer laboratories on different testing platforms. Accuracy was determined based on expected values for extracted human samples, control human samples and plasmids. The accuracy was defined as the number of correct calls vs the total number of calls made. Calls were made by the TaqMan^™^ Genotyper software for genotyping and for data points with a quality value greater than or equal to 0.95. All other data points are reported as undetermined. Calls were inspected visually and changed manually where it was observed that calls were appropriately clustered, yet the software did not make a determination. Only those calls with an expected genotype result were included. Genotype results that had an unexpected result but had no amplification (NOAMP), undetermined (UND) or invalid (INV) calls were excluded as they do not affect the accuracy of the assay. Copy number variation data were analyzed in CopyCaller^™^, where successful calls had a confidence value greater than 0.95.

#### Qualitative reproducibility

Reproducibility was analyzed by comparing the genotype or copy number variation calls for a sample over a three-day period. Extracted human samples, control human samples and plasmids were used for the genotyping assay. The ratio of concordant calls over the number of total calls was used to determine the percentage of reproducibility for each assay. NOAMP, UND or INV calls were included in the calculation. Extracted human samples and human control samples were used for the CNV assay. The ratio of concordant calls over the number of total calls was used to determine the percentage of reproducibility for each assay.

#### Minimum concentration/dynamic range

The range of input DNA that produces reproducible test results was evaluated using a dilution series of samples with final DNA concentrations ranging from 1.56 ng/μl to 100 ng/μl. The genomic DNA samples were diluted to the desired concentration. Samples that contained genetic variants included in the pharmacogenomic panel were also selected for use in the analysis. Each concentration was tested in triplicate. For the CNV assay, five extracted samples were run as a 3-point dilution series using 2-fold dilutions ranging from 10 ng/reaction, 5 ng/reaction and 2.5 ng/reaction (= 5ng, 2.5, 1.5ng/ μl). Four replicates were run per sample. Samples were analysed in CopyCaller^™^ software using the “calibrator” setting. NA17112 was used as calibrator.

#### Qualitative specificity

No template controls (NTCs) were analyzed as samples to determine the specificity for this panel. The samples named NTC were treated as control samples and not assigned a genotype by default. A total of 15 NTC samples were run across all 120 experiments.

### Allele frequencies

GenoPharm^®^ custom pharmacogenetic tool was applied to archived samples from participants in a black Zimbabwean healthy population (n = 118) and black female South African ER+ breast cancer patient cohort (n = 100). Allele and genotype frequencies for the genes tested were estimated from the results obtained. In brief allele frequency was determined for a gene of interest by dividing specific allele count by the total number of allele copies present and genotype frequency by diving the total count of genotype x by the total number of the studied population. The genotypes observed were matched to Hardy Weinberg Equilibrium (HWE) expectation. The allele frequency difference between the Zimbabwean and South African population were assessed using the Chi square (χ2) test, with a p value ≤ 0.05 indicating a significant difference throughout the population comparisons.

## Results

### Qualitative accuracy for genotyping

The qualitative accuracy was assessed by comparing the panel’s calls against calls generated from the Sanger sequencing. The average accuracy for all assays was 99.54% with a call rate of 99.41%. Separated by sample type, the breakdown for assay accuracy is as shown (Table 3). The control samples showed 99.99% concordance between the genotyping assay and Sanger sequencing method with only 2 wrong calls from a total of 15 832 tests conducted (S2 Table in S1 File). An unexpected 40% (6 out 15) non-concordance was observed for the *IFNL3* (rs12979860) assay across all the 6 concentrations tested for the extracted samples. The lowest concordance was observed at 1.56 ng//μl genomic DNA input. Concordance was 100% for the CNV assay with all the tests having a z-score less than 1.75 and confidence value greater than or equal to 0.99 (S3 Table in S1 File).

**Table 3:** Accuracy and sensitivity evaluation for the GenoPharm^®^ panel.

Sample	Variants with concordant genotypes *n (%)*	Variants with at least 1 discordant genotype *n (%)*	Accuracy
**Coriell/Plasmids (n = 43)**	119 (99.17)	1 (0.83)	99.99%
**Extracted samples (n = 15)**			
1.56 ng/μL	104 (86.66)	16 (13.33)	98.58%
3.13 ng/μL	118 (98.33)	2 (1.67)	99.58%
6.25 ng/μL	119 (99.17)	1 (0.83)	99.66%
12.5 ng/μL	119 (99.17)	1 (0.83)	99.66%
25 ng/μL	119 (99.17)	1 (0.83)	99.66%
50 ng/μL	119 (99.17)	1 (0.83)	99.66%

### Reproducibility

The reproducibility of the multiplexed pharmacogenetic panel was measured by conducting inter-run, intra-run and triplicate testing. The day-to-day and operator-to-operator overall reproducibility was 98.90%. A summary of reproducibility data analysed by sample type and concentration is shown in S4 Table in S1 File. The CNV assays had 100% reproducibility and the results are summarised in S5 Table in S1 File.

### Dynamic range

The range of DNA concentrations that would both detect consistent and reliable pharmacogenetic test results was determined. The lowest concentration which the system could detect consistently was 6.25 ng/μl. All the genotyping assays performed best at concentration between 6.25 and the manufacturer recommended 50 ng/μl. The samples with lower DNA concentrations had amplification and specificity problems (S6 Table in S1 File). A higher-than-expected number of ignored samples was also observed for those with concentrations lower than 6.25 ng/μl. The CNV assay had dynamic range of 2.5–10 ng/μl (S7 Table in S1 File). All samples, with the exception of three with higher than 2 copies, showed a confidence value of ≥ 0.99, indicating the high degree of confidence in the calls made by the software.

### Qualitative specificity

The specificity of the assays was conducted to check if the assays only react with the target sequence. A total of 1800 NTC samples were run across all the assays on the panel. Only 1 NTC sample was assigned a genotype call (S8 Table in S1 File). Therefore, the specificity of the panel was determined to be 99.94%.

### Allele frequencies

No deviation from Hardy Weinberg was observed from the studied genes. Table 4 reports allele frequencies of CYP2B6, CYP2C9, CYP2C19, CYP2D6, CYP3A4, CYP3A5 and CYP4F2 genotyped using the GenoPharm^®^ compared to allele frequencies of major ethnic groups. Allele frequencies obtained from 1000 genomes study [18], the gnomAD (version 3) study [19] and the ALFA project [20]. The allele frequencies for the Zimbabwean and South African population groups studied were highly similar, they did not show any statistically significant differences. Overall, the allelic frequencies were like those reported for Sub Saharan Africans.

**Table 4:** Comparison of allele frequencies between analysed populations (Zimbabwean and South African) and world populations.

CYP Gene	Common Alleles	ZW	SAFR	AFR	EAS	SAS	EUR	AMR
**2B6**	*1	0.4958	0.5000					
	*4	0.0242	0.0350	*^†^0.1714	*^†^0.3270	*^†^0.4400	*^†^0.0859	*^†^0.1666
	*5	0.0085	0.0100	*^†^0.0113	0.0030	*^†^0.0890	*^†^0.1123	*^†^0.0720
	*6	0.3432	0.3500	0.3744	*^†^0.2153	0.3814	*^†^0.2356	0.3732
	*18	0.1356	0.1050	0.0825	*^†^<0.010	*^†^<0.010	*^†^<0.01	*^†^0.0101
**2D6**	*1	0.3293	0.3224					
	*2	0.1159	^‡^0.2171	0.1557	^†^0.1200	*0.2900	*0.2765	*0.2208
	*2A	0.0173	0.0152	0.0063	0.0195	*^†^0.0819	*^†^0.0908	*^†^0.0859
	*4	0.0305	0.0263	0.0605	*^†^<0.010	*^†^0.1090	*^†^0.1859	*^†^0.130
	*5	0.0427	0.0329	0.0539	0.0486	0.0459	0.0295	0.0159
	*10	0.0366	0.0395	*^†^0.1127	*^†^0.5714	*^†^0.1646	*^†^0.2018	*^†^0.1484
	*17	0.2012	0.2039	0.2179	*^†^0	*^†^<0	*^†^0.0020	*^†^0.0086
	*29	0.1098	0.0855	0.1074	*^†^<0.010	*^†^<0.01	*^†^<0.01	*^†^0.0029
	*41	0.0305	0.0066	0.0182	0.0377	*^†^0.1217	*^†^0.0934	^†^0.0620
**2C9**	*1	0.8051	0.8200					
	*2	0.0042	0.0050	0.0083	0.0010	0.0348	0.1243	0.0994
	*3	0.0042	-	0.0023	0.0337	0.1094	0.0726	0.0375
	*5	0.0169	0.010	0.0166	<0.01	<0.01	<0.01	0.0014
	*6	0.0085	0	0.0109	<0.01	<0.01	<0.01	0.001
	*8	0.1356	0.1200	*^†^0.053	*^†^<0.01	*^†^0.001	*^†^0.002	*^†^0.0014
	*11	0.0297	0.0450	^†^0.0242	*^†^<0.01	*^†^0.001	*^†^0.002	*^†^0.0014
**2C19**	*1	0.7119	0.7250					
	*2	0.1356	0.1700	0.1702	*^†^0.3125	*^†^0.3579	0.1451	0.1052
	*3	0	0.0050	0.0023	*^†^0.0556	*^†^0.0123	<0.01	<0.01
	*10	0	0	0.0015	<0.01	<0.01	<0.01	0.0014
	*17	0.1525	0.100	^†^0.2352	*^†^0.0149	0.136	^†^0.2237	0.1196
**3A5**	*1	0.4492	0.5350					
	*3	0.1695	0.1650	0.1800	*^†^0.7133	*^†^0.6677	*^†^0.9433	*^†^0.7968
	*6	0.2203	0.2450	^†^0.1543	*^†^<0.01	*^†^<0.01	*^†^0.003	*^†^0.0231
	*7	0.1568	^‡^0.055	^†^0.1180	*^†^<0.01	*^†^<0.01	*^†^<0.01	*^†^0.0029
**3A4**	*1	0.2500	0.2700					
	*1B	0.7500	0.7300	0.7655	*^†^0.004	*^†^0.0399	*^†^0.0278	*^†^0.1052
**4F2**	*1	0.9492	0.9650					
	*3	0.0508	0.0350	0.0825	*^†^0.2143	*^†^0.4131	*^†^0.2903	*^†^0.2378

1 * = p<0.05 allele frequency differences major ethnic groups compared to (ZW) this study

2 ^†^ = p<0.05 allele frequency differences between major ethnic groups compared to (SAFR) this study

3 ^‡^ = p<0.05 allele frequency differences between ZW population and SAFR this study

**Fig 1:**
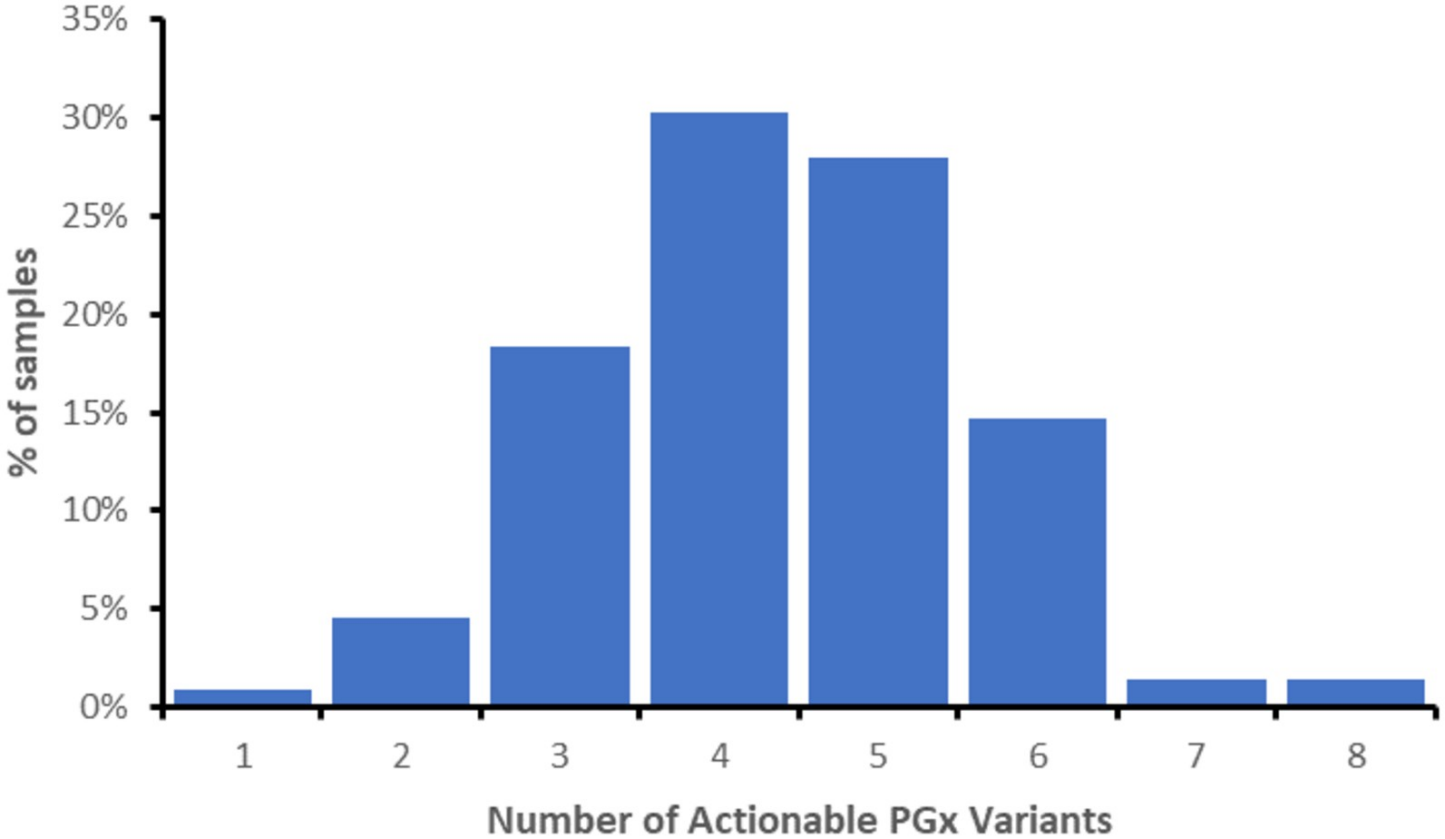
Number of pharmacogenetic variants identified from SNP genotyping using GenoPharm^®^ per sample. All of the tested 218 Black ZWL and SA populations carried at least one known actionable pharmacogenetic variant with a median of four variants. The highest number of actionable variants in a single sample was eight occurring in 0.8% of the samples.

Notable differences in allele frequencies were observed between the genotyped African population groups and the Asians, Europeans, and American populations (Table 4). CYP2B6 reduced function alleles (*6, *7, *18) had a frequency of at least 45%-48% in the African populations. The combined frequency of the reduced activity alleles *6 and *18 in this study showed statistically significant differences when compared to the allele frequencies of Asians and Europeans (p<0.0001). Eight different SNPs for CYP2C9 were interrogated and the decreased activity alleles *2, *3, *8 and *11 were observed, *8 and *11 being more prevalent in the studied African populations. The CYP2D6*17 and *29 alleles were observed at high frequencies and were the most common reduced function variants in the studied African populations. Of the 218 tested black Africans, we observed that 100% carried at least one actionable PGx variant, with a median of 4 variants in the study group as shown in (Fig 1). Amongst the actionable pharmacogenes the genes with the highest frequency of actionable phenotypes were CYP3A5, IFNL3, CYP2B6, CYP2D6 and CYP2C19 with the frequencies 77, 70, 67, 47 and 42% respectively. Results for IFNL3 must however be viewed with caution since the variant showed the least concordance in the validation tests.

## Discussion

Pharmacogenetic testing and consideration of pharmacogenetic information in clinical practice could potentially improves treatment outcome, reduces ADRs and treatment cost. Based on the increase of pharmacogenetic associations in literature, genotype guided therapy is becoming more widespread. Currently, dosing guidelines for more than 140 drugs across 31 genes have been published [16] and the FDA has included pharmacogenetic information for more than 150 drugs [21]. There is therefore need for validated PGx tests that can be used in routine clinical practice given the increased efforts to adopt use of PGx information in patient care. Single nucleotide polymorphism testing panels for pharmacogenes using PCR are important in pharmacogenomics practice to aid in clinical decision making. OpenArray^®^ technology has been widely adopted in the medical field for SNP genotyping. This study demonstrates the analytical validity and potential use of GenoPharm^®^, a custom pharmacogenetic panel which interrogates 120 variants which includes variants unique to people of African ancestry across 46 pharmacogenes encompassing 24 genes with published guidelines.

Pharmacogenomic variant frequencies can differ amongst populations with different genetic ancestries. Precision medicine’s potential can be realized with the aid of population-centered pharmacogenetic testing tools. There has been a steady increase in the validation of custom PGx panels. However most include variants that are of importance in the Caucasian and Asian populations. Comparing GenoPharm^®^ to other published panels, most custom panels do not include the African specific CYP2D6*29 and the *rs12777823* variants with potential to influence drug dosing in people of African ancestry. Lack of population-specific pharmacogenetic tests has been highlighted as one of the major draw backs in the implementation of PGx testing in research and routine practice in the African populations [22].

Our results show the validity of the test with respect to the genotypic analysis. Analytical performance of the panel was assessed by testing for performance characteristics accuracy, precision, reportable range, and specificity. The panel demonstrated an overall high genotype call rate of 99.4% comparable to the manufacturers published call rate of 99.9%. Similar high call rates have been observed in other validated custom PGx open arrays [10, 23]. Our panel was shown to be accurate, reproducible, and specific. An accuracy of 99.6%, precision of 99.3% and specificity of 99.4%. In comparison to other custom open arrays our panel had a comparable performance with respect to reported literature on accuracy (93–100%) and precision (97–100%) [10, 23–26].

Assessment of the linear dynamic range showed that for SNP genotyping testing DNA concentration greater than or equal to 6.25ng/μl produces reliable and accurate results with assay concordance of 99.66%. This is much lower than the 50ng/μl recommended by the manufacturer. Concentrations lower than 6.25 ng/μL had high level of discordant results due uneven amplification of the two alleles. We however noted a high number of no-amplification calls for sample ES2 for the different concentrations across the CYP2D6 assays. This sample had a deletion on exon 9 on the CYP2D6 gene. As expected, this also accounted for almost all the ignored calls spanning all the tested concentrations (S6 Table in S1 File). CNV assays produced passing results for samples with input DNA from 2.5, 5 and 10ng/reaction. To allow for consistency the samples should be run with the optimized DNA concentration of 10ng/reaction of DNA, However, if necessary, the results support the use of lower DNA concentrations of 2.5 and 5 ng/reaction.

Frequencies of pharmacogenetic alleles can significantly differ between ethnic and racial groups contributing to inter- population differences in drug response. This indicates the need for a tool like GenoPharm^®^ which is applicable to individuals of African ancestry. To evaluate the methodology for clinical use, the GenoPharm^®^ open array was used for SNP genotyping on two African population groups. The allele frequencies observed in the black Zimbabwean and South African population tested using GenoPharm^®^ Open array panel showed great contrast with the Asian, European and American population frequency data (Table 4). For example, CYP2D6*17 and *29 genetic variants occurred at 20% and 10% frequency respectively in contrast to reports of <0.01% in European and Asian population [27]. Our observed African specific CYP2D6*17 and *29 allele frequencies agreed with literature [14, 15, 28, 29]. The CYP2D6 allele frequencies in the two African populations cohorts tested were highly similar and comparable to the Sub-Saharan African population data. This speaks to the possible generalizability of the use of GenoPharm^®^ in the related Sub-Saharan African populations.

Further population genotyping using the panel however needs to be done, especially in Sub-Saharan populations that might have demonstrated genomic diversity in other whole exome sequencing (WES) and whole genome sequencing (WGS) studies [30, 31]. All individuals carried at least one actionable pharmacogenetic variant with published guidelines that would recommend dose adjustment or alternate therapy based on current CPIC guidelines. These findings are in line with what has been observed in other populations with nearly all individuals having at least one actionable pharmacogenetic variant [32–34]. The observed frequencies and distributions of tested pharmacogenes may have potential clinical impact. With the observed high median value of four actionable PGx variants per sample, indicative of the quantitative relevance of a PGx approach in a public healthcare setting.

The choice of variants on a panel has a direct impact on sensitivity, specificity, validity, and clinical utility of the PGx panel test [35]. The panel currently only interrogates CYP2D6 exon 9 for copy number variation. Based on the AMP PGx working group recommendations CYP2D6*5 deletion or gene duplication testing can be met by using only one location. However, this results in an inability to accurately detect conversion/ hybrid alleles without copy number measurement in the the 5’ region of the gene [9]. It is also possible to get false positive duplications especially when a CYP2D6*13 is present. Another potential limitation of this targeted pharmacogenetic panel is that it may only include variants common in specific ethnic populations. The uniqueness of this panel however lies in its ability to detect common and key variants in pharmacogenes in African populations, with the goal to utilize the panel in Africa.

PGx variants are highly dependable on the population being studied/tested, and many panels have been developed for Caucasians. For example, our panel showed high sensitivity to some key PGx variants in the African population that include the reduced function CYP2C9 variants *5, *6, *8, *11. These variants have been omitted in some of the warfarin dosing algorithms which were developed earlier [36] and should be considered in warfarin dosing for people of African ancestry. CYP2C9*8 (13%) and *11 (3%) were the common reduced function allelic variants in the African populations but not in Caucasians (Table 4). Despite the panel being not generalizable to other populations, the genotype results may be used in combination with data from other panels and available methods to generate data that can be generalized. This has potential for predicting drug-gene interactions in cases where a patient is receiving a drug metabolized by a polymorphic enzyme or are taking more than one drug.

This array covers all genes and genetic variants with actionable drug-gene-interactions (DGI) included in DPWG and CPIC guidelines. When using this method in daily practice, it will be important to consider the other drugs that patients might be taking that could influence the interpretation of drug-gene interaction predictions through phenoconversion. For example, the use of medicines that are inhibitors of CYP2D6 could result in normal metabolizers exhibiting the poor metabolizer phenotype, while the GenoPharm test would have made treatment recommendations for a normal metabolizer.

## Conclusion

GenoPharm^®^ open array panel demonstrated satisfactory performance for pharmacogenetic testing in a clinical laboratory, with high sensitivity and specificity for SNVs, small indels, and CNVs. The panel can provide clinically significant pharmacogenetic information including information of 24 genes with published dosing guidelines for individuals of African ancestry. With the analytically validated PGx array, the next step will be clinical validation of the feasibility and effectiveness of implementing PGx guided treatment in the clinical settings in Africa.

## Supporting information

S1 File Analytical validation data. (XLSX)
